# Amount of Learning and Signal Stability Modulate Emergence of Structure and Iconicity in Novel Signaling Systems

**DOI:** 10.1111/cogs.13057

**Published:** 2021-11-10

**Authors:** Vera Kempe, Nicolas Gauvrit, Nikolay Panayotov, Sheila Cunningham, Monica Tamariz

**Affiliations:** ^1^ Division of Psychology Abertay University; ^2^ Psychology Department University of Lille; ^3^ Division of Games Technology and Mathematics Abertay University; ^4^ Department of Psychology Heriot Watt University

**Keywords:** Iterated language learning, Combinatoriality, Compositionality, Iconicity

## Abstract

Iterated language learning experiments that explore the emergence of linguistic structure in the laboratory vary considerably in methodological implementation, limiting the generalizability of findings. Most studies also restrict themselves to exploring the emergence of combinatorial and compositional structure in isolation. Here, we use a novel signal space comprising binary auditory and visual sequences and manipulate the amount of learning and temporal stability of these signals. Participants had to learn signals for meanings differing in size, shape, and brightness; their productions in the test phase were transmitted to the next participant. Across transmission chains of 10 generations each, Experiment 1 varied how much learning of auditory signals took place, and Experiment 2 varied temporal stability of visual signals. We found that combinatorial structure emerged only for auditory signals, and iconicity emerged when the amount of learning was reduced, as an opportunity for rote‐memorization hampers the exploration of the iconic affordances of the signal space. In addition, compositionality followed an inverted u‐shaped trajectory raising across several generations before declining again toward the end of the transmission chains. This suggests that detection of systematic form‐meaning linkages requires stable combinatorial units that can guide learners toward the structural properties of signals, but these combinatorial units had not yet emerged in these unfamiliar systems. Our findings underscore the importance of systematically manipulating training conditions and signal characteristics in iterated language learning experiments to study the interactions between the emergence of iconicity, combinatorial and compositional structure in novel signaling systems.

## 1 Introduction

The last two decades have seen a vast expansion of experimental research into the cultural evolution of language. This research aims to understand under what conditions structured and conventionalized communication systems emerge, and how these systems change and diversify over time. At first glance, the incompatibility of language evolution and psychological testing time scales may seem to render any laboratory research that tries to address these questions futile. Yet building on seminal work by Bartlett (1932) and Esper (1966), recent methodological advances have utilized iterated learning in increasingly creative and sophisticated ways to gain insights into the fundamental principles that underpin the emergence of linguistic structure. In these iterated language learning experiments, participants are presented with novel signals for a set of meanings that they have to learn and—in some instances—use in referential communication with a partner. The results of each participant's learning and use of these novel signals are then presented to the next generation of participants along a diffusion chain. The findings have demonstrated that passing languages through an inter‐generational transmission bottleneck while also using them for intra‐generational communication leads to the emergence of artificial mini‐languages that are increasingly easier to learn and more systematically structured in ways that resemble natural languages (for a review, see Tamariz, 2017). It should be noted that compositionality can also emerge without generational transmission, for instance, during communication in large social networks (Raviv, Meyer, & Lev‐Ari, 2019) or when the predictability of referents from the context is low (Winters, Kirby, & Smith; 2018).

Although iterated language learning is an increasingly prolific line of research, it so far has focused on a limited set of learning conditions and signal spaces from which conclusions about the emergence of linguistic structure are being drawn. The current study aims to scrutinize some of these conditions to gain a fuller picture of the generalizability of what this line of research can tell us about the cognitive mechanisms that may drive language evolution. Specifically, we ask to what extent the emergence of structural features resembling those in natural languages is affected by the amount of learning, taking into account individual differences, and by modality and temporal characteristics of the signals.

## 2 Combinatorial and compositional structure

Linguistic structure emerges on two levels, which instantiate the duality of patterning considered a hallmark of human language (Hockett, 1960): On the one hand, signal spaces (also sometimes called form spaces; Cuskley, 2019) are carved up into meaningless units, which can be recombined to produce potentially unlimited sets of discrete signals. In natural languages, these units comprise the products of oral, facial, or manual gestures (e.g., phonemes or pitch contours), which are recombined in systematic ways (e.g., using phonotactic constraints), to form larger units (e.g., morphemes or words). This level of structure is referred to as *combinatorial* structure and is assumed to arise from cognitive biases that favor reuse and modification of learned building blocks (Roberts & Galantucci, 2012; Verhoef, Kirby, & De Boer, 2014). Iterated language learning experiments have explored a variety of signal spaces such as sequences of letters (Carr, Smith, Cornish, & Kirby, 2017; Cornish, Dale, Kirby, & Christiansen, 2017; Kirby, Cornish, & Smith, 2008; Kirby, Tamariz, Cornish, & Smith, 2015), syllables (Raviv & Arnon, 2018a), colors (Cornish, Smith, & Kirby, 2013), tones (Kempe, Gauvrit, Gibson, & Jamieson, 2019) as well as drawn squiggles (Tamariz & Kirby, 2015), tracings on a continuously moving pad (Galantucci, Kroos, & Rhodes, 2010), abstract graphical forms called Ferros (Cuskley, 2019), whistled pitch contours (Verhoef, Roberts, & Dingemanse, 2015; Verhoef et al., 2014), and manual gestures (Motamedi, Schouwstra, Smith, Culbertson, & Kirby, 2019). These experiments have shown that both inter‐generational transmission (Verhoef et al., 2014) and referential communication (Little, Eryılmaz, & De Boer, 2017b) can lead to the emergence of combinatorial structure. The combinatorial signal units that emerge in these experiments are shaped by the specific affordances of each signal space. For example, color sequences are transformed into chunks of reusable color combinations (Cornish et al., 2013), unfamiliar squiggles are transformed into assemblies of familiar geometrical or graphical elements (Tamariz & Kirby, 2015), and complex melodies are transformed into concatenations of shorter pitch contours (Verhoef et al., 2014; see Cuskley, 2019, for a recent overview).

On the other hand, when signals refer to meanings, the combinatorial units that make up individual signals are mapped onto dimensions of the meaning space in systematic ways. Recombination of such mappings allows for unlimited productivity in the expression of meanings. The mappings can be non‐iconic and non‐motivated but are systematic in the sense that the link between a set of combinatorial units and a meaning dimension is used consistently. This level of structure is referred to as *compositional* structure, in analogy to the morpho‐syntax of natural languages where sublexical and lexical units such as phonemes, morphemes, and words systematically map onto syntactic and semantic features. Compositionality also involves positional structure (Galantucci & Garrod, 2011) such that the ordering of signals conveys dimensions of meaning, often in a hierarchical manner (Saldana et al., 2019). For example, in iterated learning experiments where signals comprise strings of letters, certain morpheme‐like letter combinations can become associated with a referent's color, while others may map onto a referent's shape or direction of movement, and it does not matter which particular letter combinations map onto each of these meaning dimensions as long as these mappings are consistent (Kirby et al., 2008, 2015).

However, in natural languages, the specific links between features of the signals and dimensions of the meaning space are not entirely arbitrary but exhibit some degree of iconicity. In oral languages, iconicity frequently, but not exclusively, involves *conventional sound symbolism* (Cuskley & Kirby, 2013) where some features of the signal space, for example, certain sound clusters, are systematically associated with certain meaning dimensions. More generally, such non‐arbitrary relationships involve a degree of homeomorphism between the topologies of the signal and meaning spaces (Little et al., 2017b), a relationship that has also been termed *relative iconicity*. For example, both in natural languages and artificial signaling systems, longer signals tend to be associated with bigger referents or more complex meanings (Kempe et al., 2019; Lewis & Frank, 2016; Little et al., 2017b). Furthermore, words with similar sounds tend to have similar meanings (Monaghan, Lupyan, & Christiansen, 2014; Tamariz, 2008), and letters with similar shapes tend to map onto similar sounds (Jee, Tamariz, & Shillcock, 2018). In sign languages, *sensory sound symbolism* is more frequent as the relationship between signals and meanings tends to involve some degree of physical resemblance. For example, in many sign languages, signals bear visual similarity with the objects or actions they denote (Perniss, Thompson, & Vigliocco, 2010), and a considerable number of oral languages possess ideophones, that is, words like the Japanese *“gorogoro”* denoting multiple heavy objects rolling or the Siwu *“pumbuluu”* denoting an enormous round belly (Dingemanse, Blasi, Lupyan, Christiansen, & Monaghan, 2015), where form features like reduplication or dimensions of vowel quality depict meaning dimensions in analogous ways, a phenomenon also termed *absolute iconicity* (Little, Eryilmaz, & De Boer, 2017a, 2017b).

Referential communication studies in which participants were asked to memorize or negotiate a set of novel signals showed that for some signaling systems, for example, drawings or gestures, absolute iconicity can emerge right from the start (Fay, Ellison, & Garrod, 2014; Fay, Lister, Ellison, & Goldin‐Meadow, 2014; Motamedi et al., 2019), and the iconic signals subsequently become simplified and conventionalized with repeated use (Lister & Fay, 2017). For other signaling systems like Leap Motion (Little et al., 2017b) or binary tone sequences (Kempe et al., 2019), where the physical resemblance between signal and meaning space is limited, the emergence of relative iconicity requires transmission over several generations as it takes time for users to find suitable and well‐motivated mappings between signal features and relevant meaning dimensions, for example, using signal duration as an indicator of referent size.

### 2.1 Manipulating the transmission bottleneck

The notion of a transmission bottleneck refers to limitations on the amount and kind of input that learners encounter and the constraints on processing the input. In natural language acquisition, children encounter a subset of their ambient language and generalize the structural regularities they are able to extract from it. In iterated language learning experiments, the poverty of the stimulus found in natural language acquisition is sometimes recreated by withholding a proportion of signal‐meaning pairings from training, and then testing participants on the full set (e.g., Kirby et al., 2008; Motamedi et al., 2019). In other studies, where learners encounter the entire signaling system designed for a specific experiment (e.g., Kempe et al., 2019; Kirby et al., 2015), memory and processing limitations are assumed to drive the addition of structure to aid learning (Cornish et al., 2017).

Such different operationalizations of the transmission bottleneck in iterated language learning experiments make it difficult to pinpoint specific constraints that drive the emergence of structure. First, it is unclear which specific input manipulations best represent input constraints that operate in natural language acquisition. Some studies only present about 50% of signals (e.g., Kirby et al., 2008) to encourage generalization during testing, while other studies present the entire signaling system (e.g., Carr et al., 2017; Kirby et al., 2015). This procedural difference may matter because randomly selected subsets of signals may or may not contain whatever structural regularities are starting to emerge over the course of transmission. Natural language acquisition often involves exposure to a special register—child‐directed speech—that has been shown to emphasize relevant structural features of a language (Kempe & Brooks, 2005; Savickiene, Kempe, & Brooks, 2009) and is therefore likely to facilitate the discovery of structural regularities. Thus, withholding parts of the input in iterated language learning experiments may inadvertently constrain or bias structure discovery in ways that are not well‐controlled and are different from the way natural input aids language acquisition.

Second, iterated language learning experiments show considerable variation with respect to how often individual signals are presented during training. For example, in Kirby et al. (2008, 2015), a relatively small set of signals was presented six times each, while in Carr et al. (2017) or Saldana et al. (2019), much larger sets of signals were presented three times each. Again, methodological feasibility seems to determine the overall amount of training, but these different training regimens make it difficult to disentangle the effects of presentation frequency from effects of structural complexity. Even for identical numbers of categories and meaning dimensions, the number of repetitions is likely to affect encoding. For example, frequent exposure to individual signals might encourage rote‐memorization thereby preventing learners from extracting structure. If learners subsequently notice discrepancies between training and novel testing items, they may “invent” new signals based on pre‐existing linguistic knowledge (but see Hartley & Fedzechkina, 2020, for recent evidence that the native language does not always exert an influence on structure addition). Less frequent exposure to individual signals, on the other hand, might encourage structure discovery and generalization.

Finally, structure addition is also affected by individual differences between learners, the effects of which on iterated learning are difficult to disentangle from effects of input selection and training regimen. While some accounts have suggested that poor learners, that is, learners with limited processing capacity such as children, are more likely to add structure to aid learning (Hudson Kam & Newport, 2005), recent evidence shows that structure addition is driven by strong learners who are better able to extract regularities from the input (Johnson, Siegelman, & Arnon, 2020). However, it is not clear whether more structure addition by strong learners results from advantages in the encoding of information or the representation of structural regularities, and how either of these advantages may impact inter‐generational transmission.

The first aim of the present study was therefore to manipulate the amount of learning while taking into account individual differences in learning capacity by introducing fixed learning criteria. Participants had to reach either a more stringent or a more lenient accuracy threshold in comprehension and production of the training signals before being allowed to proceed to the testing phase. Rather than simply varying the amount of exposure to individual signals, this manipulation ensured that participants learned the input to the same degree regardless of individual differences in the amount of learning trials needed to achieve the same level of performance. We predicted that if the amount of learning facilitates structure extraction, then the combinatorial and compositional structure should emerge more readily when learners had to reach a more stringent criterion. If, on the other hand, the imposing structure serves primarily as an aid to learning, combinatorial and compositional structure should be more likely to emerge in the lenient training condition.

#### 2.1.1 Manipulating modality

The emergence of structure may be affected not just by constraints that arise from the amount of learning but also by the specific characteristics of signals and meanings. Typically, participants in iterated language learning experiments are presented with printed labels for a set of meanings (but see Carr et al., 2017, for experiments that provided additional synthesized auditory input and Smith & Dediu, 2020, for experiments transmitting spoken pseudo‐words) and are asked to produce signals by typing letters on a keyboard. The letter strings presented at the outset of learning largely obey phonotactic and graphotactic constraints that are familiar to literate participants. Such a signal space does not permit to study the emergence of duality of patterning because the basic combinatorial units—graphemes—and the rules for how to combine them are already provided. Moreover, a comparison of learners that were either familiar or unfamiliar with musical notation has shown that musical literacy per se greatly facilitated the ability to reproduce compositional structure (Tamariz, Brown, & Murray, 2010) suggesting that, by analogy, structure emergence in the domain of language may have been over‐estimated in studies that use printed word‐like letter strings as signals for literate participants.

A number of studies have explored the emergence of combinatorial structure using unfamiliar signal spaces such as whistled pitch contours (Verhoef et al., 2014), color sequences (Cornish et al., 2013), or Ferros (Cuskley, 2019). However, without linking such signals to meanings, these studies were restricted to exploring combinatorial structure in isolation from the compositional structure, which is bound to paint an incomplete picture of how linguistic structure can emerge on multiple levels. To study the emergence of the duality of patterning, novel signaling systems that do not contain pre‐specified familiar combinatorial units need to be combined with meanings. Studies that have used unfamiliar signals like Leap Motion‐generated sounds (Eryilmaz & Little, 2017; Little et al., 2017a, 2017b), whistled pitch contours (Verhoef et al., 2015), or tracings on a moving pad (Del Giudice, 2012; Galantucci et al., 2010; Roberts & Galantucci, 2012; Roberts, Lewandowski, & Galantucci, 2015) to represent a range of meaning spaces have shown that emergence of structure interacts with the iconic affordances of each signal space: The greater the potential for iconicity of the signal space, the longer it takes for combinatorial and compositional structure to emerge. A frequent observation is that visual signals such as manual gestures appear to have greater iconic potential (Little et al., 2017b; Perniss et al., 2010), presumably because, in contrast to vocal gestures, manual gestures possess more degrees of freedom thereby providing greater capacity for homeomorphic mappings onto a wide range of meaning dimensions. Yet when the number of meaning dimensions exceeds the number of signal features—a constellation that is more typical for vocal gestures—combinatorial and compositional structure emerge more readily (Little et al., 2017b). Thus, when signals are unfamiliar, iconic mappings are encouraged and the differences between the manual or articulatory gesture repertoires that are available to produce visual or auditory signals can determine modality differences in affordances for iconicity.

In addition, signal modality is often confounded with temporal stability. Many visual signals, such as letter strings or drawings, remain accessible over much longer time periods than auditory signals. Even manual gestures used in natural sign languages comprise temporal changes on a slower time scale than vocal gestures where relevant contrasts rely on changes in the tens of milliseconds. Differential sensitivity to temporal characteristics may be responsible for the attested modality‐specificity in statistical learning (Frost, Armstrong, Siegelman, & Christiansen, 2014): Regularities extracted in one modality do not reliably transfer to another (Redington & Chater, 1996; Tunney & Altman, 1999). Moreover, within individuals, there is no correlation across modalities in the capacity to extract regularities (Siegelman & Frost, 2015). Finally, developmental trajectories of statistical learning abilities also seem to be modality‐specific with continuous improvement with age found in the visual but not the auditory modality (Raviv & Arnon, 2018b). Such modality‐specific differences in statistical learning arise because similar computational principles operate over different neural networks that are characterized by somewhat differing processing and maturation constraints: The auditory cortex displays greater sensitivity to temporal information, while the visual cortex is more sensitive to spatial information and less capable of temporal accumulation of information over extended periods of time (Chen & Vroomen, 2013). Accordingly, different temporal presentation rates have opposite effects on statistical learning in the auditory and the visual domain (Emberson, Conway, & Christiansen, 2011).

It is thus unclear to what extent findings from the iterated language learning experiments that use temporally stable visual signals generalize to oral languages, which comprise rapidly fading auditory signals. On the one hand, the “Now‐or‐Never bottleneck” created by the temporal transience of auditory input imposes the need for rapid chunking to pass the information on for further processing at various levels of abstraction (Christiansen & Chater, 2016), a process that underpins the need for structure creation. Under this account, extracting or imposing structure for the purposes of re‐representation at various hierarchically organized levels of processing serves to ameliorate the rapid loss of sensory information associated with temporal transience. Indeed, when rapid fading was imposed on visual signals in an iterated language learning experiment, combinatorial structure emerged more readily than when the same visual signals were temporally stable (Galantucci et al., 2010). On the other hand, rapidly fading auditory signals may be more taxing on working memory capacity than temporally stable visual signals and may thus restrict the capacity for structure discovery. In line with this idea, iterated language learning experiments that use spoken pseudo‐words show greater variability in structure emergence than what has been observed for written pseudo‐words (Smith & Dediu, 2020). Moreover, for children, who have limited working memory capacity, iterated learning experiments failed to find evidence for the emergence of structure when using transient auditory binary tone signals (Kempe et al., 2019) while the use of temporally more stable gestural signs did provide some evidence for the emergence of structure (Bohn, Kachel, & Tomasello, 2019). However, it is difficult to compare these findings on structure emergence in children because temporal features were confounded with signal familiarity as the binary tone signals in Kempe et al. (2019) were less familiar than the gestures used in Bohn et al. (2019). It should be noted that structure did not emerge either in children when unfamiliar pseudo‐words presented via a temporally stable syllable bank in Raviv and Arnon (2018a) underscoring the importance of separating temporal characteristics of signals from their familiarity in iterated language learning studies.

Thus, in addition to examining the role of amount of learning in iterated language learning experiments in Experiment 1, Experiment 2 aimed to disentangle effects of signal modality from effects of temporal stability on structure emergence by presenting learners with stable versus fading visual signals. Moreover, because the visual signals in Experiment 2 had the same information‐carrying capacity as the auditory signals in Experiment 1, we were able to perform a direct modality comparison of fading signals from both experiments to see whether structure emerges more readily in the auditory domain when controlling for temporal characteristics of the signals.

### 2.2 The present study

Below we report on two iterated language learning experiments in which participants were arranged into 12 chains of 10 generations each and had to learn to associate 4–6‐bit binary sequences with eight meanings that differed in shape (spiky or rotund), size (large vs. small), and brightness (dark vs. light). The binary sequences were chosen because they constitute signals that are unfamiliar to participants to control for effects of prior knowledge and pre‐existing biases. While this signal space may seem impoverished if one perceives it to consist only of two combinatorial elements, its richness emerges from the possibilities of combining the two elements into small chunks such as bigrams (e.g., 00, 11, 01, 10) or trigrams (e.g., 000, 010, 111, etc.), which, in turn, can be linked to the dimensions of the meaning space to create compositional structure. The training comprised both comprehension and production tasks. In the comprehension task, participants were presented with one signal at a time and had to select the associated meaning from the set of eight referents. In the production task, participants were presented with one referent at a time and had to produce the associated signal. In both experiments, training lasted as long as it took participants to either comprehend or produce a pre‐specified number of signals accurately. During testing, participants had to produce all signals again but without feedback. Finally, we also gave participants a self‐comprehension task where half of the signals they themselves had produced during testing were presented for comprehension.

In Experiment 1, we manipulated the amount of learning. Participants learned binary tone sequences comprising low and high tones and had to complete as many rounds of alternating comprehension and production training as it took them to use either three (*Short Training condition*) or five (*Long Training condition*) signal‐meaning mappings correctly over the course of two comprehension‐production blocks. These particular criteria were based on prior research using binary signals that demonstrated that these are difficult signals to learn so that imposing a criterion of more than five correct mappings would have proven infeasible. Imposing different learning criteria implies that participants who had to reach a more stringent criterion had less opportunity to modify signals in the training phase than with a more lenient criterion. However, in both conditions, participants had the same opportunity to modify signals in the testing phase. Thus, a more stringent criterion served to increase the amount of learning which, as we show below, involved a larger amount of exposure.

In Experiment 2, we manipulated the temporal dynamics of signal presentation by comparing iterated learning of binary color sequences in a *Fading condition* where individual blue or orange circles either appeared and faded one by one at the same rate as the tones in Experiment 1 with a *Stable condition* where all circles were presented simultaneously and remained visible for the same duration it had taken to present a fading sequence of the corresponding length. To ensure comparability to the auditory signals, Experiment 2 employed the same training regimen as the *Short Training condition* of Experiment 1, that is, participants had to achieve three correct signal‐meaning mappings in two comprehension‐production rounds before being allowed to proceed to testing. Finally, we provide a direct modality comparison by comparing the *Short Training condition* using binary tone sequences from Experiment 1 with the *Fading condition* using binary visual sequences from Experiment 2. This comparison allows us to disentangle the effects of modality from effects of temporal stability on the emergence of structure. Both experiments received ethical clearance from Abertay University and were conducted online.

In both Experiments, we measured learnability (operationalized as trials to criterion), transmission fidelity, combinatorial structure, compositional structure and one instantiation of iconicity, the association between sequence length, and referent size, which in previous research with binary signals had been identified as the preferred form of iconicity for this signal‐meaning space (Kempe et al., 2019) rather than associations between pitch and shape or pitch and brightness. Because the two experiments overlap in many features and because the modality comparison involves conditions from each of the experiments, we describe and discuss the experiments jointly to facilitate comparison of the effects of amount of learning, of temporal dynamics, and of signal modality on the emergence of iconicity and structure.

## 3 Method

### 3.1 Participants

*Experiment 1*: One hundred and twenty student participants above the age of 18 years were recruited via social media and through the Abertay SONA system. We had hoped for the task to be sufficiently engaging to spread on social media but were able to recruit only about 10 participants using this method. As a result, most participants received course credit for participation.

*Experiment 2*: One hundred and twenty participants above the age of 18 were recruited through the crowd‐sourcing website, Prolific Academic, and paid £4.00 for participation. The change in recruitment strategy was necessary as Experiment 1 had exhausted the available naïve participant pool of this small university.

### 3.2 Materials

#### 3.2.1 Meanings

**Fig. 1:**
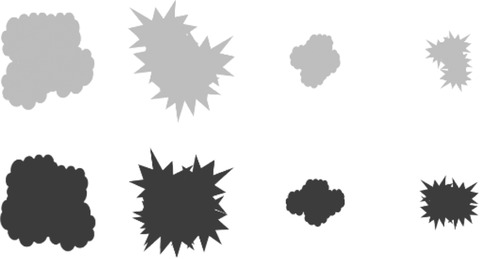
Meanings differing in size (large vs. small), shape (spiky vs. rotund), and brightness (light vs. dark gray)

In both experiments, the meaning space comprised eight visual images of abstract shapes differing in shape (spiky *kiki*‐type vs. round *bouba*‐type), size (small shapes occupied half the size of large shapes), and brightness (light gray vs. dark gray). All referents also had unique properties based on their particular shapes such that while all spiky shapes had contours comprising acute angles, the actual arrangement of these spikes differed from shape to shape (see Fig. 1).

*Experiment 1*: The auditory signals consisted of 500‐ms sine‐wave tones (high tone: 440 Hz = musical note *a*; low tone: 293.7 Hz = musical note *d*), which were synthesized and concatenated with an inter‐tone‐interval of 500 ms. A set of eight sequences was created by randomly combining the two tones such that half of the sequences were four, and the other half six tones long to introduce some variability in signal duration in order to encourage variability in the duration of participants’ reproductions and to maintain compatibility with previous studies (Kempe et al., 2019).

*Experiment 2*: The visual signals comprised sequences of blue and orange dots. In the *Fading condition* (described in the Procedure section for Experiment 2), dots were revealed one by one from left to right with each dot fading away once the next dot to the right of it had appeared. The inter‐dot onset interval was 1000 ms mimicking the interval between the onset of tones in Experiment 1. The fading animation lasted 400 ms and started simultaneously with the onset of the next dot in the sequence. In the *Stable condition* (described below), all the dots in a sequence were revealed simultaneously and remained on screen for a total time determined by multiplying the number of dots in the sequence by 500 ms.

### 3.3 Procedure

The experiment was implemented for web browsers using HTML5 technologies to be compatible with a variety of devices, including desktops, laptops, smartphones, and tablets. After accessing the weblink, participants viewed an instruction screen explaining that they would learn a two‐tone (Experiment 1) or a two‐color (Experiment 2) language to label some unfamiliar shapes, that correct comprehension and production of the artificial “words” would earn them points and that they had to collect a certain number of points.

Unlike in other iterated language learning experiments, where participants are exposed to signal‐meaning pairs before the training phase commences, here they started with a comprehension training block. In effect, from the very beginning, participants had to guess the meanings of unfamiliar binary sequences, which was designed to encourage the induction of motivated signal‐meaning links. On each trial, the participant first saw a display of all eight meanings dispersed on random locations across the screen, combined with either auditory or visual presentation of the signal. Participants were presented with the signals one at a time and had to select the object they thought it referred to. If they chose the correct object, that object was then surrounded by a green circle and participants earned a point, which was displayed on their scoreboard on the screen. If they chose a wrong object, it was surrounded by a red circle while the correct object was surrounded by a green circle, and their score remained unchanged. Immediately after that, all objects except the correct one encircled in green faded away. The correct object remained visible on the screen, and the associated sound sequence was played to provide feedback. For the next trial, all objects reappeared on the screen in a different spatial configuration, and participants received the next signal to try to identify its meaning. After completing eight trials presenting all eight signals, participants moved on to the production block. If participants had correctly identified the pre‐specified number of meanings, they received a star.

The production training block always followed the comprehension training block. A trial started with one of the eight objects being presented in the middle of the screen. Participants were asked to produce a sound (Experiment 1) or color (Experiment 2) sequence to label the object. Once participants had decided that they had finished creating a sequence, they clicked on the “next” button that triggered a “correct” or “wrong" prompt to indicate whether their input had matched the learned signal or not. They then were presented with the correct signal before proceeding to the next trial. Participants completed all eight trials in random order, gaining a point every time they correctly produced the trained sequence. If they had correctly reproduced the pre‐specified number of signals, a star appeared on the scoreboard.

If during the comprehension and production training blocks, participants had not gained two stars, they underwent further comprehension and production training by going through further rounds. This set‐up made it possible to reach the pre‐specified learning criterion exclusively for correct comprehension, exclusively for correct production, or for correct trials in both tasks combined. Successfully collecting two stars completed the training phase.

The production testing block started after a short chime to refocus the participants’ attention. It followed the same procedure as the production training block, except no feedback was given, as the “correct” sequences were not presented to the participants. Unbeknown to the participants, their input in this block was used as the training input for the next participant in the chain. To prevent degeneration of the language into a set of ambiguous signals, participants were alerted when they had entered a previously produced sequence and were told to retry until they had created a unique signal for each meaning. The self‐comprehension testing block followed immediately after the production testing block. It was identical to the comprehension training block except that participants were told that they would be presented with the signals that they themselves had created in the previous block to test how well they were able to “understand themselves.” No item‐level feedback was given about the accuracy of their individual choices, and only four randomly selected objects were presented to prevent a strategy of selecting objects by elimination of previously chosen objects.

As we had initially hoped for the experiment to spread on social media, we ended it with a brief questionnaire asking how participants had found out about it and asking them to rate their enjoyment of participation on a 5‐point Likert scale. We also provided a comment box to allow participants to include any feedback they would like to give before being debriefed. The subsequent debriefing screen explained the rationale behind the experiment and showed participants the percentage of correct trials they had achieved in the self‐comprehension testing block. A detailed description of the web implementation is provided in the Supplementary Materials at https://osf.io/2uqma/.

*Experiment 1*: Participants were randomly assigned to the *Short* or *Long Training condition* and told that they had to reach a certain number of points (three in the *Short Training condition*; five in the *Long Training condition*) per training round to obtain a star and that they needed two stars to proceed to the final testing round. Next, participants saw a screen with two buttons marked with an “up” and a “down” arrow arranged next to each other and were invited to press them to familiarize themselves with the high and low tones. The position (left vs. right) of the buttons was counterbalanced across participants. In order to receive the auditory sequences in the comprehension block, participants were prompted to press a button labeled “listen.” In order to generate the auditory sequences in the production block, participants were asked to use the on‐screen arrow buttons located underneath the presented meaning. Each button press elicited a high or low 500‐ms buzzing tone depending on the arrow, and participants could string as many tones together as they wanted. The average duration of Experiment 1 was 27.5 min, ranging between 8 and 128 min depending on how long it took participants to reach the learning criterion.

*Experiment 2*: Participants were told they would learn a two‐color language. Instead of buttons with “up” and “down” arrows, participants pressed an orange or a blue button arranged and counterbalanced in the same way as the arrow buttons in Experiment 1. In the comprehension block, participants were prompted to press a button labeled “show word” to reveal the sequence of color circles. In the production block, participants pressed the colored buttons successively to create the color sequences. In both tasks, circles appeared following the temporal constraints of each condition: in the *Fading condition*, any previous color circle disappeared over the course of 400 ms as a new one was entered by a button click. In the *Stable condition*, all circles remained visible until participants pressed the “next” button. As in the *Short Training condition* of Experiment 1, to receive a star, participants had to score three correct trials per round comprising a comprehension or production block. The production and self‐comprehension testing blocks were presented once participants had scored two stars. The average duration of the entire experiment was 22.3 min, ranging between 6 and 70 min depending on how long it took participants to reach the learning criterion.

## 4 Results

**Fig. 2:**
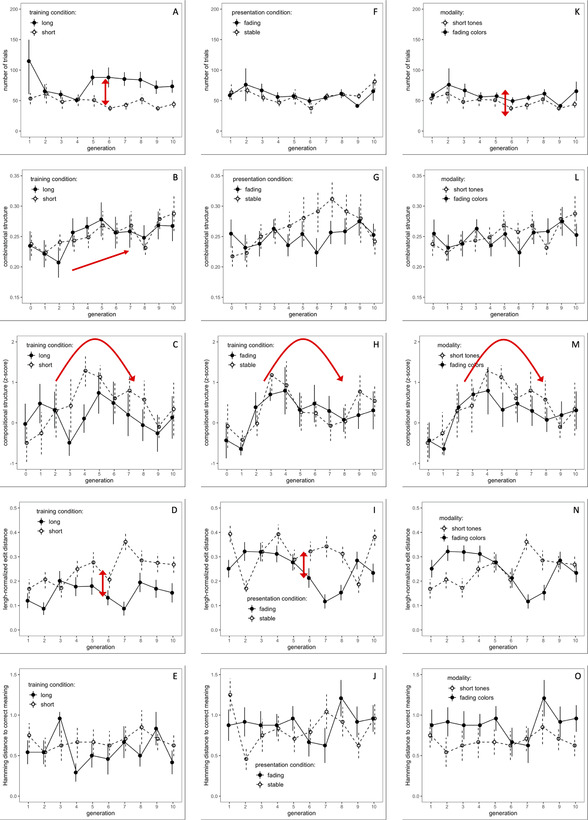
Generational change in the number of trials (Panels A, F, K), combinatorial structure (Panels B, G, L), compositional structure (Panels, C, H, M), edit distance of produced signal to previous generation signal (Panels, D, I, N), and Hamming distance of selected meaning to target meaning in self‐comprehension (Panels E, J, O) for auditory tone sequences in Experiment 1 (left panels), visual color sequences in Experiment 2 (middle panels), and modality comparison (right panels). Error bars indicate ± 1 *SEM*. Red arrows indicate the direction of significant effects.

All data were subjected to growth curve analysis with R (Version 3.6.3; R Core Team, 2020) using the *lme4* R‐package (Bates, Mächler, Bolker, & Walker, 2015). The statistical significance of each term was evaluated using the Satterthwaite approximation implemented in the *lmerTest* R‐package (Kuznetsova, Brockhoff, & Christensen, 2017). We fitted linear mixed‐effects models with fixed effects of *Generation* (centered, including linear and quadratic effects following Beckner, Pierrehumbert & Hay, 2017) and *Condition* (sum‐coded) as well as their interaction, which are presented in Table 1. For system‐level dependent variables (*Number of Trials to Criterion*, *Combinatorial Structure*, and *Compositional Structure*), we included random intercepts and slopes of *Generation by Chain*. For the item‐level dependent variables (*Edit Distance*, *Hamming Distance*, and *Sequence Length*), we additionally included random effects of individual meanings and implemented the maximal random effect structure that would allow for model convergence (Barr, Levy, Scheepers, & Tily, 2013). All data and analyses are available in the Supplementary Materials at https://osf.io/2uqma/. Table 1 and Fig. 2 combine the presentation of results of both experiments as well as for the modality comparison using the same model structure for each dependent variable across experiments to facilitate comparison.

**Table 1:** Parameter estimates of growth curve analyses fitted to the different dependent variables in Experiments 1 and 2 as well as for the direct comparison between short training auditory sequences from Experiment 1 and fading visual sequences from Experiment 2

Dependent Variable	Intercept	Gen	Gen^2^	Cond	Genx Cond	Gen^2^ x Cond
Experiment 1: Long Versus Short Training of Auditory Signals (Tone Sequences)						
Trials to criterion^1^	64.295***	–54.461	36.898	15.257*	11.964	19.474
Combinatorial structure^1^	0.252***	0.160*	–0.026	–0.001	–0.006	–0.040
Compositional structure^1^	0.283	0.693	–2.914*	–0.135	–1.186	1.927
Edit distance^2^	0.198***	0.732	–0.398	0.048*	–0.456	0.148
Hamming distance^2^, ^a^	0.625***	0.154	0.154	–0.049	–0.151	0.045
Experiment 2: Stable Versus Fading Visual Signals (Color Circle Sequences)						
Trials to criterion^1^	60.059***	‐37.960	78.600*	–0.025	–23.437	–43.240
Combinatorial structure^1^	0.257***	0.133	–0.091	–0.008	–0.067	0.133
Compositional structure^1^	0.266*	1.620	–2.157*	–0.041	0.020	–2.156
Edit distance^2^	0.280***	–0.552	0.206	–0.031*	–0.470	0.138
Hamming distance^2^, ^a^	0.856***	0.414	1.109	0.022	0.206	–0.070
Modality Comparison: Short Training Auditory Signals Versus Fading Visual Signals						
Trials to criterion^[Link]^, ^b^	54.536***	–63.911	26.392	–5.498*	–0.502	–8.968
Combinatorial structure^1^	0.251***	0.116	0.028	0.002	0.050	‐0.015
Compositional structure^1^	0.322	1.760	–3.937*	0.097	0.119	–0.904
Edit distance^2^, ^a^	0.248***	0.083	–0.101	–0.001	–1.105	0.445
Hamming distance^2^	0.778***	0.247	0.362	–0.101	0.238	0.561

*Note*. ^1^model *lmer* notation in R: Dependent Variable ∼ Cond * poly(Gen, 2) + (poly(Gen, 2) | Chain)

^2^ model *lmer* notation in R: Dependent Variable ∼ Cond * poly(Gen, 2) + (poly(Gen, 2) | Chain) + (Cond * poly(Gen, 2) | Meaning

^a^ Model did not converge, so we removed the random effect of the quadratic term of Gen by Meaning

^b^ Model did not converge so we retained only the linear random slope of Generation

*** *p* < .001

* p < .05, Gen = *Generation*, Cond = *Condition*.

### 4.1 Learnability

A number of trials to criterion served as an indicator for how easy it was to learn the signal‐meaning links. Analyzing this indicator allowed us to perform a manipulation check for Experiment 1 to see whether imposing the more stringent criterion did indeed result in longer training as we had intended. In addition, this measure can reveal whether learnability improved over the course of transmission as would be expected based on previous research on iterated language learning (e.g., Kirby et al., 2008, 2015).

*Experiment 1*: We found the main effect of *Condition* showing that, as expected, participants completed on average 78.1 trials to reach the more stringent learning criterion, compared to an average of 47.9 trials, to reach the more lenient criterion thus justifying labeling the two conditions as *Long* vs. *Short Training*. There was no evidence that the learnability of the links between auditory sequences and meanings changed over the course of transmission (see Fig. 2, Panel A).

*Experiment 2*: As the learning criterion was identical in the *Fading* and the *Stable conditions*, the number of training trials would only differ between these conditions if one temporal presentation mode was inherently more difficult than the other. The analysis revealed that this was not the case nor was there any evidence that the learnability of links between color sequences and meanings changed over the course of transmission (Fig. 2, Panel F).

#### 4.1.1 Modality comparison

The main effect of *Condition* (*Short Training Auditory* vs. *Short Training Visual Fading*) suggests that participants required fewer trials overall with auditory signals (47.9 trials on average), compared to visual signals (58.7 trials on average), to reach the learning criterion of three correct responses in two training rounds (Fig. 2, Panel K).

### 4.2 Combinatorial structure

Determining combinatorial structure for a set of binary sequences that make up a signaling system is challenging because the specific subcomponent strings that participants might be recombining when producing different sequences are not known. For example, two sequences like 0010 and 0100 can comprise the bigrams 01, 10, and 00 or the single 0 and the trigram 010, and it is not clear whether the bigram 00 has been reused across sequences or the trigram 010. We measured combinatorial structure using a combinatorial coefficient (CC) inspired by the *form recombination index* (Galantucci et al., 2010) and the minimum description length (MDL) principle (Barron, Rissanen, & Yu, 1998): For a given language (i.e., a set of signals) such as L = {0010, 0100, 0011, 0101}, we determine all possible *n*‐grams and check for the shortest one that would give the shortest overall description. For instance, the bigram A = 01 leads to the recoding of L as {A:01, 0A0, A00, 0A1, AA}. While the original language L was 16 elements long, the recoded language is reduced to a length of 15 elements, counting only alphanumeric symbols. Thus, compared with all other possible *n*‐grams, 01 is the shortest one that leads to the best reduction. The MDL principle, therefore, suggest that 01 is an elementary unit. Scanning all possible *n*‐grams again, we find that no other *n*‐gram would lead to a shorter recoding of L so that we keep {A:01, 0A0, A00, 0A1, AA} as the best‐compressed version of L. Depending on the language, the elementary units determined in this way may be used in several signals. To quantify how much the elementary units tend to be reused, we use the *form recombination index* on the best‐compressed version of L, returning CC. Hence, CC(L) as our measure of combinatoriality evaluates the propensity of elementary units to be used across different signals in L. The R‐function used to compute the CC is provided in the Supplementary Materials available at https://osf.io/2uqma/.

*Experiment 1*: The significant linear effect of *Generation* provides evidence for the emergence of combinatoriality over the course of transmission. Neither the effect of *Condition* nor the interaction reached significance suggesting that combinatorial structure emerged regardless of the required amount of learning (see Fig. 2, Panel B).

*Experiment 2*: The analysis yielded no significant effects (Fig. 2, Panel G).

#### 4.2.1 Modality comparison

The analysis yielded no significant effects (Fig. 2, Panel L).

### 4.3 Compositional structure

Following the procedure first outlined in Kirby et al. (2008), we determined compositional structure for the sets of signals produced at testing by correlating similarities between all pairs of signals, measured as length‐normalized Levenshtein edit distances (described in more detail in the section on transmission fidelity below), with similarities between the corresponding pairs of meanings, measured as Hamming distances, and determined the *z*‐score of this correlation within a distribution of 1000 random permutations of signal‐meaning pairings (Mantel, 1967).

*Experiment 1*: The significant negative quadratic effect of *Generation* indicated a curvilinear trajectory of the emergence of compositional structure, which increased in the earlier generations before gradually falling again in the later generations (Fig. 2, Panel C).

*Experiment 2*: As in Experiment 1, the negative quadratic effect of *Generation* provided evidence of a curvilinear rise‐and‐fall trajectory of compositional structure (Fig. 2, Panel H).

#### 4.3.1 Modality comparison

For the short auditory and fading visual signals, the negative quadratic effect of *Generation* provided evidence for the increase and subsequent decrease of compositional structure (Fig. 2, Panel M).

Note that in all conditions, the mean *z*‐scores of the Mantel test never exceeded 1.96, the level of chance at *p* = .05, although some of the individual *z*‐scores in some of the chains did (see Appendix A).

### 4.4 Transmission fidelity

To ascertain transmission fidelity, we measured the reproduction accuracy of signals by computing length‐normalized Levenshtein edit distances between signals produced for the same meaning during testing in consecutive generations. This measure takes account of the number of insertions, deletions, and substitutions required to transform one sequence into another, divided by the length of the longer sequence. The higher the values, which can range from 0 to 1, the less accurately a sequence is reproduced. If transmission fidelity increases this measure should decrease over the course of transmission.

*Experiment 1*: We found a main effect of *Condition*, indicating that edit distance was overall lower (mean length‐normalized edit (nLED) = 0.23), and hence transmission fidelity was higher when participants underwent more training to reach a more stringent learning criterion before testing, compared to less training (mean nLED = 0.32). (Fig. 2, Panel D). There was no linear or quadratic effect of *Generation* suggesting that fidelity of signal reproduction did not change over the course of transmission.

*Experiment 2*: We found a main effect of *Condition*, indicating that edit distance was overall lower, and hence fidelity higher in the *Fading condition* (mean nLED = 0.32) than in the *Stable condition* (mean nLED = 0.37). As in Experiment 1, there were no linear or quadratic effects of *Generation* (see Fig. 2, Panel I).

#### 4.4.1 Modality comparison

The analysis yielded no significant effects (see Fig. 2, Panel N).

### 4.5 Self‐comprehension

While edit distances indicate the accuracy of signal reproduction, we also checked whether there was an improvement in self‐comprehension, that is, whether participants were able to correctly identify the meaning of their own signals produced during testing. To this end, we computed Hamming distances to identify whether a referent selected during self‐comprehension differed from the target referent in one, two, or all three dimensions and subjected the average Hamming distance out of three as our measure of self‐comprehension to the statistical analyses.

*Experiment 1*: There was no indication for a difference in mean Hamming distances between conditions (*Short*: 0.67, *Long*: 0.57), nor for any change in self‐comprehension over the course of transmission (see Table 1 and Fig. 2, Panel E).

*Experiment 2*: As in Experiment 1, there was no difference in mean Hamming distances between conditions (*Fading*: 0.89, *Stable*: 0.83), nor was there evidence for a change in comprehension over the course of transmission (Fig. 2, Panel J).

#### 4.5.1 Modality comparison

As for Experiments 1 and 2, there was no difference in mean Hamming distances between conditions (*Short*: 0.67, *Fading*: 0.89), nor was there evidence for a change in comprehension over the course of transmission (Fig. 2, Panel O).

### 4.6 Iconicity

To determine whether participants formed an iconic association between meaning size and signal length as in previous studies with the same signal space (Kempe et al., 2019), we fitted growth curve analyses using a full model with fixed effects of *Generation*, *Condition*, and *Size* and random effects of chains and individual meanings using a full random effect structure to signal length as the dependent variable. The parameter estimates and associated *p*‐values are given in Table 2.

**Table 2:** Parameter estimates of growth curve analyses fitted to signal length in Experiments 1 and 2 as well as for the direct comparison between short training auditory sequences from Experiment 1 and fading visual sequences from Experiment 2 using the model: Signal Length ∼ Size * Condition * poly(Generation,2) + (Size * poly(Generation,2) | Chain) + (Condition * poly(Generation,2) | Meaning). Where models failed to converge random slopes of interactions between either *Size* or *Condition* and either the linear or quadratic term were removed (for specific models see Supplementary Materials)

Fixed Effect	Audio (Tones) Experiment 1	Visual (Color Circles) Experiment 2	Audio Versus VisualExp. 1 VersusExp. 2
Intercept	4.589***	4.613***	4.455***
Generation	–6.802*	–7.950*	–7.357*
*Generation* ^2^	1.084	0.645	2.794
*Size*	–0.165	0.158	0.269*
*Condition*	–0.197	–0.096	–0.063
*Size* x *Generation*	3.474	–0.924	3.484
*Size* x *Generation* ^2^	–2.052	–4.143	–3.066
*Condition* x *Generation*	0.418	–0.457	–0.136
*Condition* x *Generation* ^2^	–2.714	1.146	–1.004
*Size* x *Condition*	–0.296*	–0.081	–0.191
*Size* x *Condition* x *Generation*	–3.007	1.411	–2.997
*Size* x *Condition* x *Generation* ^2^	2.512	2.575	1.498

*Note*. ^***^ *p* < .001

* *p* < .05.

**Fig. 3:**
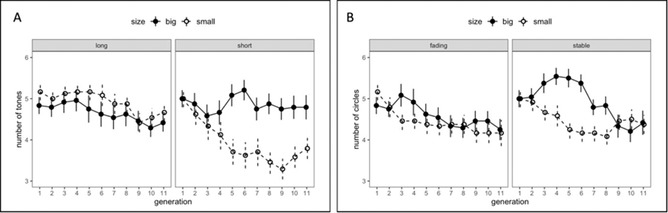
Length of signals for large and small meanings as a function of training condition for auditory sequences in Experiment 1 (Panel A) and of presentation condition for visual color sequences in Experiment 2 (Panel B). Error bars indicate ± 1 *SEM*.

*Experiment 1*: The results showed that signals became shorter over the course of transmission. The significant interaction between referent size and condition suggests that in the *Short Training condition*, signals for small meanings were consistently shorter than those associated with large meanings (see Fig. 3, Panel A).

*Experiment 2*: As in Experiment 1, the significant effect of *Generation* indicates signals became shorter over the course of transmission (see Fig. 3, Panel B).

#### 4.6.1 Modality comparison

The significant effect of *Generation* indicates that signals became shorter over the course of transmission (compare the right graph in Panel A and the left graph in panel B of Fig. 3). The significant effect of *Size* indicates that signals for small referents were overall shorter than signals for big referents.

### 4.7 General discussion

In this study, we asked whether the amount of learning, temporal characteristics, and modality of unfamiliar signals affect the emergence of combinatorial and compositional structure as well as of iconicity in iterated learning of unfamiliar binary signals. Experiment 1 manipulated the amount of learning of binary auditory sequences using a stringent versus lenient accuracy criterion resulting in longer versus shorter training phases. Experiment 2 manipulated temporal decay by presenting binary visual sequences in either a stable or a fading condition, with the latter matching the temporal characteristics of the auditory sequences of Experiment 1. A direct comparison between the *Short Training condition* of Experiment 1 and the *Fading condition* of Experiment 2 allowed us to perform a direct modality comparison between auditory and visual signals independently of the amount of learning and temporal characteristics. Below, we will discuss these three factors that we set out to examine in this study in turn.

### 4.8 Amount of learning

The manipulation check for Experiment 1 confirmed that imposing a stringent criterion induced longer training as indicated by a larger number of trials participants needed to reach the criterion in the *Long Training condition*, compared to the *Short Training condition*. This manipulation affected the emergence of relative iconicity: Only in the *Short Training condition*, but not in the *Long Training condition*, did participants systematically associate sequence length with referent size. Iconicity can provide an initial solution to the problem of linking unfamiliar signals to meanings (Lister & Fay, 2017) by exploiting salient and transparent associations that are easier to remember and—in the context of communicative situations—may constitute shared knowledge (Sulik & Lupyan, 2018). While the present experiment did not include communication, in the *Short Training condition*, where the amount of learning was constrained by limited exposure and limited opportunity for rote‐memorization, the pressure to retain signal‐meaning links facilitated an association between length and size such that signals for small referents became much shorter than signals for big referents, and this length difference increased over the course of transmission. Note that while in the *Short Training condition*, there was more opportunity for learning biases to affect the modification of signals, participants in the *Long Training condition* were also able to modify signals when reaching the testing block. However, at this point, they would have undergone longer training and engaged in more rote‐memorization, which presumably restricted exploration of the signal space with respect to its affordances for potential cross‐modal mappings to the meaning space. Thus, when communicative pressure is absent, constraints on the amount of learning can drive the emergence of iconicity.

On the other hand, combinatorial structure, that is, the reuse of subsequences as combinatorial units or building blocks, emerged in both learning conditions regardless of the amount of learning. In contrast to Verhoef, Kirby, and de Boer (2016), we did not find a delay of the emergence of combinatoriality in the condition that favored iconicity, which in this case was the *Short Training condition*. However, while Verhoef et al. (2016) varied iconic affordances, we varied the amount of learning and found that it only affected the emergence of iconicity but not the emergence of combinatoriality.

The amount of learning also had no effect on the emergence of compositional structure, which in both learning conditions increased intermittently only to decrease again in later generations. There was no evidence that this trajectory was different when there was greater opportunity to rote‐memorize as in the *Long Training condition*. We will return to this point below.

Fidelity of signal reproduction did not increase over the course of transmission: When presented with a specific meaning, it did not become easier for participants to produce the corresponding signal nor did it become easier for them to identify the meaning when presented with a signal they had just produced. However, we also found that overall transmission fidelity was higher in the *Long Training condition*, compared to the *Short Training condition*, but only for production and not for self‐comprehension. An asymmetry between production and comprehension performance is in line with findings from Little et al. (2017b) where Leap Motion was used to signal geometrical shapes, and performance in signal recognition was lower than in signal reproduction. However, here the difference between conditions in the accuracy of signal reproduction might have in part been an artifact of the difference in signal length that arose from the emergence of length‐size iconicity in the *Short Training condition*: The same number of deviating elements between two sequences will result in lower length‐normalized edit distances for longer than for shorter sequences. To the extent that sequences were longer in the *Long Training condition*, transmission fidelity might have been overestimated in that condition. Given that the dimensionality of the meaning space was identical between conditions, similar artifacts in the computing of Hamming distances can be ruled out for comprehension. Thus, we cannot be sure whether the greater transmission fidelity in the *Long Training condition* was due to a greater amount of learning or production of longer signals.

### 4.9 Temporal characteristics

We had hypothesized that the temporal characteristics of the signal presentation can affect the emergence of structure. In Experiment 2, we manipulated whether signals were stable or fading at a rate similar to the auditory presentation of Experiment 1, while ensuring an equal amount of training in both conditions by imposing the same learning criterion. However, there was no effect of these temporal characteristics of the signals on the combinatorial or compositional structure. In fact, for the visual signals, we did not find any evidence for the emergence of combinatoriality at all suggesting that—all things being equal—chunking in the visual modality may emerge less readily than in the auditory modality and may be governed by different principles such as the Gestalt principles of visual perception (Wertheimer, 1923, 1958) or operate on different spatial frequencies not comparable to temporal chunking in the auditory modality.

As with the auditory signals of Experiment 1, compositional structure emerged in a transient fashion raising across earlier generations but declining subsequently, a trajectory that will be discussed below. At this point, it is important to note that this curvilinear trajectory of the emergence of compositionality did not differ between stable and fading visual signals.

While temporal characteristics of the signals had no effect on combinatorial or compositional structure, we found that during testing, the fading signals were reproduced with greater fidelity than the stable ones. Previous evidence suggests that statistical learning is superior in the auditory modality at faster presentation rates of about 350 ms and in the visual modality at slower presentation rates of 750 ms (Emberson et al., 2011). The presentation rate of 500 ms used in the *Fading condition* lies between these values, and it is not clear whether this rate is slow enough to provide an advantage for learning in the visual domain. We can therefore only speculate that the *Fading condition* may have removed interference between dot configurations that could have arisen when presenting the colored binary dots simultaneously in the *Stable condition* and suggest that this unexpected finding is in need of replication.

Temporal characteristics had no effect on the emergence of length‐size iconicity in the visual domain. Although sequences became shorter over the course of transmission, there was no statistical evidence for different rates of change according to size. One possibility, to be explored in further research, is that intra‐modal associations (e.g., associating the size of one visual image with the length of another one) do not provide an additional learning advantage.

As with the auditory signals of Experiment 1, learnability did not improve over the course of transmission of visual signals either. Even though the signals became progressively shorter, this increase in production efficiency was not associated with commensurate gains in learning the signals for specific meanings. This is in contrast to many previous iterated language learning studies and suggests that transmission fidelity relies on structure as a memorization aid. The transient emergence of compositional structure observed in these experiments was apparently insufficient for participants to create the stable, enduring links between signal features and meaning dimensions that could have facilitated learning.

### 4.10 Modality effects

Learning conditions and temporal characteristics being equal, auditory signals required fewer trials to criterion than visual signals suggesting that they were easier to learn than visual signals. Although this preliminary finding needs further corroboration through careful manipulation of temporal characteristics of the signals using a design that segregates participant variability from modality‐induced variability, it is in line with evidence for modality‐specificity in statistical learning that reflects the predisposition of the auditory cortex for rapid temporal integration (Chen & Vroomen, 2013; Siegelman & Frost, 2015).

There was no modality effect on the emergence of combinatorial structure, suggesting that the shorter amount of training used for modality comparison was insufficient for the combinatorial structure to emerge and that the emergence of combinatoriality observed for the auditory sequences in Experiment 1 must have been mainly carried by the *Long Training condition*. The present design did not allow us to determine whether a greater amount of learning would have lead to the emergence of combinatorial structure in the visual domain as well. For now, all we can conclude is that with sufficient exposure, combinatorial structure can emerge in unfamiliar auditory signals.

No modality difference was found with respect to the trajectory of the emergence of compositionality, which mirrored the curvilinear trajectory already identified for each modality separately. No modality difference was found either with respect to the emergence of iconicity: In both conditions, shorter sequences were used to signal smaller referents. Recall that in the auditory domain, iconicity emerged only when learning was limited. The main effect of size in the temporally transient auditory and visual signal suggests that this tendency toward establishing iconic signal‐meaning links may be independent of modality although it seemed to be much weaker for visual signals.

Taken together, the evidence discussed so far suggests that constraints on the amount of learning may favor iconicity as a solution to the creation of motivated signal‐meaning links. We should note that the slightly different recruitment strategies in the experiments (predominantly students gaining course credit in Experiment 1 vs. online participants gaining payment in Experiment 2) may limit comparability in subtle ways. Still, given that a length‐size association was evident in both modalities when the amount of learning was constrained and signals were fading, it would appear that the emergence of iconicity benefitted from less learning and less temporal stability of the signals confirming its role as a first‐pass strategy to establish motivated signs under difficult learning conditions. While future research should explore further why rote‐learning limits exploration of iconic affordances, our results demonstrate that subtle variations in learning conditions and signal presentation rate can affect the emergence of iconic mappings.

### 4.11 Compositional structure

We found a rise‐and fall‐pattern of compositional structure in both modalities, which was not affected by the amount of learning or temporal characteristics suggesting that the propensity to try to establish structured signal‐meaning links is quite robust even if the outcome is not always successful as indicated by the fact that the *z*‐scores obtained through the Mantel test only exceeded chance on a few occasions. Previous research has shown that the emergence of compositionality is strongly supported by communicative pressure: Signal‐meaning links need to be systematic enough so that they can be recovered by an interlocutor who is trying to identify a meaning communicated to them (Kirby et al., 2015; for a review see Smith, 2018). It could be argued that without a communicative task, there was insufficient pressure for compositionality in these experiments. However, some iterated language learning experiments have demonstrated that compositionality can emerge without communication when ambiguity filters are used (e.g., Kirby et al., 2008), just as we did in these experiments: Whenever participants attempted to produce an ambiguous signal, that is, a signal they had already used, they were alerted to this and asked to produce another signal. What we found was that compositional structure increased somewhat over the first few generations but then started to decrease again toward the end of the transmission chains. Appendix A provides examples of binary sequences that had the highest degree of compositionality within each condition, followed by the subsequent signal system in the chain in which part or all of this compositional structure was lost again. What these examples show is a quite remarkable similarity in structure induction for the dimensions that were not expressed iconically: Shape was usually coded on the first position of the binary sequences attesting to its salience as a perceptual dimension that is being privileged for labeling (e.g., Landau, Smith, & Jones, 1988), while brightness was often coded on the second or third position of the sequence. The fundamental difficulty that a learner has with compositional signals of this kind is that in the absence of clear conventionalized combinatorial structure, it is very difficult to identify which elements are systematically linked to meaning dimensions, given the many degrees of freedom afforded by the remaining elements, which introduce a considerable amount of noise. A combinatorial structure that, for example, establishes bigrams as the basic units would make it easier to identify systematic variation at the beginning of the second bigram. However, if such combinatorial units have not been established yet, identifying the positions that carry the compositional structure during comprehension is extraordinarily difficult, especially for longer sequences, so that systematicity introduced by one learner during production can easily be missed by the next one during comprehension.

This account leaves unanswered the question as to why compositionality seemed to increase over the first few generations—after all, if identifying which elements of the sequence carry the structure is difficult, one would expect continuous fluctuations in compositionality to arise. We speculate that while identifying regularities in one or two signals early on in the transmission chain may still be feasible and learners may be able to build upon this, tracing the regularities across all signals of the system is difficult in the absence of a clear combinatorial structure that guides the learner to where to look for such regularities.

One could argue that one obvious solution for compositionality in binary sequences would have been to create a perfect compositional system that maps 3‐bit‐sequences onto a 2 × 2 × 2 meaning space. Yet such a minimalist compositional system never emerged in any of the 240 signaling systems produced in both experiments. One possibility is that single tones and single color circles did not have enough perceptual salience to serve as morpheme‐like compositional units. Another possibility is that such a minimalist system violates the duality of patterning as the constituent components would cease to be meaningless building blocks. Future research should explore the criteria that guide the inter‐relation between combinatorial and compositional units.

Many previous iterated language learning experiments were not designed to explore the relationship between the emergence of combinatoriality and compositionality because they either supplied combinatorial units from the outset, like graphemes and associated phonemes as the building blocks of pseudo‐words (e.g., Kirby et al., 2008, 2015) or omitted meanings altogether when presenting color sequences (Cornish et al., 2013), consonant letter sequences (Cornish et al., 2017), slide whistles (Verhoef, 2012; Verhoef et al., 2014), or Ferros (Cuskley, 2019). As we argued above, such signal spaces are unlikely to capture the entirety of constraints that govern the emergence of the duality of patterning. The relationship between the emergence of combinatorial and compositional structure can only be explored when unfamiliar signals are paired with unfamiliar meanings. One problem with the unfamiliar signal spaces that have been used so far to express meaning, for example, physical or digital slide whistles (Verhoef et al., 2016, 2015), tracings on a moving digitizing pad (Del Giudice, 2012), or Leap Motion sound controllers (Eryilmaz, & Little, 2017; Little et al., 2017b), is that it is difficult to quantify combinatorial and compositional structure as the signals comprise continuous change over time on a range of parameters, for example, pitch or spatial location, and it is unclear how to segment continuous signals into constituent building blocks. Although interesting inroads into computing combinatorial structure have been made using hidden Markov models to analyze either hand coordinates or amplitude and frequency values of auditory signals created with Leap Motion controllers (Little et al., 2017b), we suggest that the simple binary sequences used here constitute a promising signaling system that offers an opportunity to explore and quantify combinatorial and compositional structure in tandem.

Although there was evidence for the emergence of combinatoriality and iconicity in some conditions and transient increase of compositionality in all conditions, our experiments found no increase in learnability over the course of transmission despite the signals generally getting progressively shorter. This shows that learnability is driven by the transparency and systematicity of signal‐meaning links, which, in the absence of prior experience with a signaling system, are difficult to forge and remain fragile. Even the length‐size association or the compositional elements that emerged were insufficient to allow for reliable signal reproduction due to the many degrees of freedom that arise from the lack of combinatorial structure. For example, when attempting to label a small referent, participants may have produced shorter sequences, but shortening alone did not sufficiently specify all features of the target sequence. Consequently, when being presented with their own shorter sequences in the self‐comprehension task, signal length per se would not have provided participants with sufficient information to be able to select the correct referent among the four small ones. Similarly, coding spikiness with a low tone in the first position was insufficient information for memorizing the rest of the sequence. Thus, our findings suggest that both combinatoriality as a way to induce the internal structure of signals and iconicity as a way to establish initial signal‐meaning links may constitute the pre‐requisites for the emergence of compositional structure.

In summary, our results underscore the importance of examining the emergence of combinatorial and compositional structure in tandem and to systematically explore the parameter space that is defined by signal familiarity, signal modality, temporal characteristics of signals, and the specifics of the training regimen, to better understand how the various processing constraints and their interaction can affect the evolution of language.

### Open Research Badges

This article has earned Open Data and Open Materials badges. Data are available at https://osf.io/2uqma/ and materials are available at https://osf.io/2uqma/.

## Appendix A

### A.1

Signaling systems with peak levels of compositional structure within each condition, and their successor signaling systems in the subsequent generation, illustrate the decline in structure responsible for the curvilinear relationship indicated by the negative quadratic term in the statistical models. In the binary sequences, 0 represents high tones in the auditory language and orange color in the visual language. Bold‐face marks meaning dimensions that are being marked and underline is used to highlight one of the values of a binary meaning dimension in the signals. “Correct” = accuracy with respect to the signal from the previous generation; nLED = length‐normalized edit distance to the signal from the previous generation.

Condition: *Long Training* (*Auditory*), Chain 5

**Table:**

#	Gen 5, *z* = 2.28	Length	Size	Shape	Brightness	Gen 6, *z* = 2.00	Correct	nLED
1	**0** 1 **0** 0	4	Big	**Fluffy**	**Light**	**0** 1 **0** 0	Yes	0
2	**1**1 **0** 00	**5**	**Big**	**Spiky**	**Light**	**1**1 **0** 00	Yes	0
3	**0** 0 **0** 1	4	Small	**Fluffy**	**Light**	**0** 0 **0** 1	Yes	0
4	**1**1 **0** 0	**4**	**Small**	**Spiky**	**Light**	**1**1 **0** 0	Yes	0
5	**0** 0**1**11	5	Big	**Fluffy**	**Dark**	**0** 0**1**11	Yes	0
6	01**1**00010	**8**	**Big**	Spiky	**Dark**	01**1**00010	Yes	0
7	**0** 0011111	8	Small	**Fluffy**	Dark	10001	No	0.625
8	**1**1**1**0001	**7**	**Small**	**Spiky**	**Dark**	**1**1**1**0001	Yes	0

*Note*. For Generation 5, the shape is systematically coded in the first position in all sequences except # 6, brightness is coded systematically in Position 3 in all sequences except #7; size is systematically coded by sequence length within each brightness level for the spiky but not the fluffy meanings.

Condition: *Short Training* (*Auditory*), Chain 7

**Table:**

#	Gen 3, *z* = 3.27	Length	Size	Shape	Brightness	Gen 4, *z* = 2.73	Correct	nLED
1	**1** 0 **0** 001	**5**	**B ig**	**F luffy**	**L ight**	**1** 0 **0** 10	No	0.333
2	**0**1 **0** 01	**5**	**B ig**	**Spiky**	**L ight**	**0**1 **0** 01	Yes	0
3	**1** 0 **0** 1	**4**	**Small**	**F luffy**	**L ight**	**1** 0 **0** 1	Yes	0
4	**0**1 **0**	**3**	**Small**	**Spiky**	**L ight**	**0**1 **0**	Yes	0
5	**1** 1**1**111	**6**	**B ig**	**F luffy**	**Dark**	**1** 1**1**111	Yes	0
6	**0**1**1**111	**5**	**B ig**	**Spiky**	**Dark**	**0**1**1**11	No	0.167
7	**1** 1**1**1	**4**	**Small**	**F luffy**	**Dark**	**1** 1**1**1	Yes	0
8	**0**1**1**1	**4**	**Small**	**Spiky**	**Dark**	**0**1**1**1	Yes	0

*Note*. For Generation 3, the shape is systematically coded in the first position in all sequences, brightness is coded systematically in Position 3 in all sequences; size is systematically coded by length within each shape/brightness combination in all sequences.

Condition: *Stable* (*Visual*), Chain: 104

**Table:**

#	Gen 5, *z* = 3.75	Length	Size	Shape	Brightness	Gen 6, *z* = –1.15	Correct	nLED
1	**0** 1 **0** 110	**6**	**B ig**	**F luffy**	**L ight**	11010	No	0.333
2	**1**1 **0** 10	5	Big	**Spiky**	**L ight**	1110	No	0.2
3	**0** 1 **0** 11	**5**	**Small**	**F luffy**	**L ight**	11101	No	0.6
4	**1**1 **0** 11	5	Small	**Spiky**	**L ight**	11011	Yes	0
5	**0** 1**1**01	**5**	**B ig**	**F luffy**	**Dark**	110101	No	0.333
6	**1**1**1**01	**5**	**B ig**	**Spiky**	**Dark**	1111	No	0.2
7	**0** 1**1**1	**4**	**Small**	**F luffy**	**Dark**	1011	No	0.5
8	**1**1**1**1	**4**	**Small**	**Spiky**	**Dark**	1101	No	0.25

*Note*. For Generation 5, the shape is systematically coded in the first position in all sequences, brightness is coded systematically in Position 3 in all sequences; size is systematically coded by the length in all sequences within each shape/brightness combination except for sequences #2 and #4.

Condition: *Fading* (*Visual*), Chain: 109

**Table:**

#	Gen 4, *z* = 3.00	Length	Size	Shape	Brightness	Gen 5, *z* = –0.21	Correct	nLED
1	1 **1**	2	Big	Fluffy	**L ight**	11	Yes	0
2	1 **1** 0101	6	Big	Spiky	**L ight**	11010	No	0.167
3	0 **1** 101	5	Small	Fluffy	**L ight**	0010	No	0.5
4	0 **1** 0110	6	Small	Spiky	**L ight**	10010	No	0.5
5	**00**100	5	Big	**F luffy**	**Dark**	0001010	No	0.428
6	**1**10010	6	**B ig**	**Spiky**	Dark	110101	No	0.333
7	**00**0101	6	Small	Fluffy	**Dark**	00100	No	0.6
8	**1**1010	5	**Small**	**Spiky**	Dark	1010010	No	0.428

*Note*. For Generation 4, the shape is systematically coded in the first position for the dark meanings; brightness is coded systematically in Position 2 in all sequences except #6 and #8. There is no iconic representation of size via length.
